# Cross-Protection Induced by Encephalitis Vaccines against COVID-19 Might be a Reason for Relatively Lower Mortality Rate in Some Countries

**Published:** 2020-04-21

**Authors:** Shojiro Katoh, Toshihiko Obayashi, Jegatheesan Saravana Ganesh, Masaru Iwasaki, Senthilkumar Preethy, Samuel JK Abraham

**Affiliations:** 1 Edogawa Evolutionary Lab of Science, Edogawa Hospital Campus, 2-24-18, Higashi-Koiwa, Edogawa, Tokyo 133-0052, Japan.; 2 Edogawa Hospital, 2-24-18, Higashi-Koiwa, Edogawa, Tokyo 133-0052, Japan.; 3 Waikato District Health Board, Hamilton 3204, New Zealand.; 4 Yamanashi University-Faculty of Medicine, 1110, Shimokato, Chuo, Yamanashi 409-3898, Japan.; 5 The Fujio-Eiji Academic Terrain (FEAT), Nichi-In Centre for Regenerative Medicine (NCRM), PB 1262, Chennai 600034, Tamil Nadu, India.; 6 The Mary-Yoshio Translational Hexagon (MYTH), Nichi-In Centre for Regenerative Medicine (NCRM), PB 1262, Chennai 600034, Tamil Nadu, India.

**Keywords:** COVID-19, immunity, heterologous, encephalitis, vaccines, japanese encephalitis vaccines

## Abstract

COronaVIrus Disease 2019 (COVID-19) is an on-going pandemic attributed to a novel virus named SARS‐CoV‐2. Comparing the statistics of incidence and death rates between nations reveals that there is discrepancy amongst countries in these regards, even between countries that share borders. We herein present information from the literature indicating how cross-protection against COVID-19 conferred by the encephalitis vaccine could be the reason for lower fatality rate in the countries where immunization against encephalitis is widespread or included in national programs. This may pave the way for arriving at efficient prevention strategies as well as vaccine development.

COVID-19, an acronym for COronaVIrus Disease 2019, previously 2019‐nCoV, is an on-going pandemic attributed to a novel virus that belongs to the coronavirus (CoV) family, SARS‐CoV‐2. As of April 20th, 2020, the infection has spread to 210 countries with a total of 2,314,621 confirmed cases and 157,847 deaths worldwide, according to the World Health Organization (WHO) (https://covid19.who.int/). The CoVs are a large family of single-stranded RNA viruses (+ssRNA) that can cross species barriers and cause illnesses in humans ranging from the common cold to more severe diseases such as Middle East respiratory syndrome (MERS) and severe acute respiratory syndrome (SARS) (1). Coronaviruses were first described in 1966 by Tyrell and Bynoe who named them based on the crown-like morphology of the spherical virion with surface projections that resemble a solar corona. Among the four CoV sub-families (alpha‐, beta‐, gamma‐, and delta‐coronaviruses), SARS‐CoV‐2 belongs to the B lineage of beta‐CoV and is closely related to the SARS‐CoV. The widely reported initial clinical sign for case detection is pneumonia but gastro-intestinal and asymptomatic infections are also being reported in children (2). 

With the death toll increasing daily, we considered the mortality rates reported by the European Centre for Disease Prevention and Control (3) as of April 20^th^, 2020 wherein reported deaths are provided in parentheses after a country’s name: Africa: the five countries reporting most deaths are Algeria (375), Egypt (239), Morocco (141), South Africa (54) and Cameroon (42). Asia: the five countries reporting most deaths are Iran (5,118), China (4,636), Turkey (2,017), Indonesia (582) and India (543). America: the five countries reporting most deaths are United States (US) (40,682), Brazil (2,462), Canada (1,580), Mexico (686) and Ecuador (474). Europe: the five countries reporting most deaths are Italy (23,660), Spain (20,453), France (19,718), United Kingdom (16,060) and Belgium (5,683). Oceania: the four countries reporting most deaths are Australia (70), New Zealand (12), Guam (5) and Northern Mariana Islands (2). Interestingly, countries like the US, Spain and Italy have more fatalities than China, the epicentre of the outbreak with a much bigger population. In addition to several factors such as the preparedness of countries like South Korea and Japan due to their earlier fight against SARS and MERS, the emphasis these countries put on developing *in vitro* diagnostics and therapeutics against the disease (4), co-operation of the public (5), small but significantly contributing factors such as sense of Life Worth Living (Ikigai) in Japan (which actually has been proven to decrease the risk of mortality (6)) etc., which potentially decreased fatalities in these countries compared to Italy, Spain, USA etc., cross-protection against COVID-19 conferred by the encephalitis vaccine also seems to be a key parameter worthy of further investigation. This connection is well illustrated by comparing the large number of fatalities in Italy with the small number of deaths in their neighbouring country, Austria (452 deaths) (3), where, unlike Italy, encephalitis vaccination is included in its national immunization schedule (7). This hypothesis is further supported by several additional reports. 

Viral-induced encephalitis is associated with four different kinds of viruses i. Arboviruses (e.g., eastern equine encephalitis, Japanese encephalitis (JE), La Crosse encephalitis, St. Louis encephalitis, western equine encephalitis, West Nile virus encephalitis), ii. Enteroviruses (e.g., coxsackievirus, polioviruses), iii. Herpes viruses and iv. Other viruses such as measles, mumps, and rubella, which cause secondary encephalitis (8). Of these, JE is the leading cause of viral encephalitis worldwide, with an estimated 50,000 cases and 15,000 deaths annually. It is a member of the genus Flavivirus, which also includes the dengue, yellow fever and West Nile viruses and is transmitted by Culex mosquitoes. In northern Europe and northern Asia, flaviviruses causing encephalitis have evolved to use ticks as vectors because of their abundance in cooler climates and hence the encephalitis caused by flavivirus is termed tick-borne encephalitis (9). It is interesting to note that CoVs, though primarily recognized as respiratory pathogens, are also encephalitis-inducing infectious agents; especially, HCoV-229E, HCoV-OC43, and SARS-CoV, which possess neuroinvasive properties and their viral RNA has been detected in human brains. A few years after the 2002-2003 SARS-CoV epidemics, associated viral particles were identified in the brain tissue of infected individuals with central nervous system (CNS) issues associated with oedema, neurodegeneration, and gliosis. The viruses accessed the brain through the olfactory bulbs, where they infected neuronal cells, and induced a lethal disease in an area of the body with restrained immune infiltration (8). Immunization with inactivated JE vaccine (INV) and live attenuated vaccine (LAV) induces a cross-immune response that confers cross-protection against dengue viruses (DENVs) (10). Tripartite motif-containing (TRIM) proteins are active players in antiviral innate immunity and overlapping molecular determinants govern the antiviral activities of TRIM56 against infections by yellow fever virus (YFV), dengue virus serotype 2 (DENV2) and human CoV (HCoV-OC43) (11). The neuroinvasive properties of SARS-CoV2 may also underlie respiratory failure in patients with COVID-19 (12). Furthermore, a case of meningitis was recently attributed to COVID-19 in Japan (13).

Next, we considered countries where JE immunization is widespread or included in national programs namely, Japan, Laos, Malaysia, Nepal, South Korea, Thailand, Sri Lanka, and Vietnam. Again, in all of these countries, the fatality rate due to COVID-19 is very low (3) compared with countries that don’t immunize against JE. In China, the epicentre of the COVID-19 outbreak where the JE vaccine is included in its national immunization schedule, the fatality rate is 2.3% compared to 7.3% in Italy where the JE vaccine is not routinely administered (14).

Therefore, the implications and applications of the immunological cross-protection conferred by the JE vaccine ought to be considered in design and development of vaccines against COVID-19 as well as other antiviral therapeutic approaches, so that development of efficient and effective CoV vaccine and therapy is ensured. 
